# Regulation of angiogenesis and cancer cell proliferation by human vault RNA1-2

**DOI:** 10.1093/narcan/zcaf028

**Published:** 2025-08-30

**Authors:** Stefano Gallo, Anastasiia Suspitsyna, Daniel Sanchez-Taltavull, Rafael Sebastián Fort, Maria Ana Duhagon, Deborah Stroka, Norbert Polacek

**Affiliations:** Department of Chemistry, Biochemistry and Pharmaceutical Sciences, University of Bern, Freiestrasse 3, 3012 Bern, Switzerland; Graduate School for Cellular and Biomedical Sciences, University of Bern, 3012 Bern, Switzerland; Department of Chemistry, Biochemistry and Pharmaceutical Sciences, University of Bern, Freiestrasse 3, 3012 Bern, Switzerland; Graduate School for Cellular and Biomedical Sciences, University of Bern, 3012 Bern, Switzerland; Department of Visceral Surgery and Medicine, Inselspital, Bern University Hospital and University of Bern, 3010 Bern, Switzerland; Sección Genómica Funcional, Facultad de Ciencias, Universidad de la República, Montevideo 11400, Uruguay; Departamento de Genómica, Instituto de Investigaciones Biológicas Clemente Estable, Montevideo 11600, Uruguay; Unidad Académica de Genética, Facultad de Medicina, Universidad de la República, Montevideo 11800, Uruguay; Department of Visceral Surgery and Medicine, Inselspital, Bern University Hospital and University of Bern, 3010 Bern, Switzerland; Department of Chemistry, Biochemistry and Pharmaceutical Sciences, University of Bern, Freiestrasse 3, 3012 Bern, Switzerland

## Abstract

Noncoding RNAs play pivotal roles in tumorigenesis and cancer progression. Recent evidence has identified vault RNAs (vtRNAs) as critical regulators of cellular homeostasis. The human genome encodes four vtRNA paralogs, which are differentially expressed in cancer tissues and contribute to tumor development. The best studied vtRNA1-1 is involved in regulating apoptosis resistance, autophagy, lysosomal biogenesis, and drug resistance. Here, we present the first comprehensive characterization of vtRNA1-2 using a knockout hepatocellular carcinoma (HCC) cell line. Loss of vtRNA1-2 impaired cancer cell viability and proliferation by modulating mitogen-activated protein kinase signaling. Additionally, vtRNA1-2-deficient cells exhibited reduced motility and a decreased invasive potential. Unlike vtRNA1-1, vtRNA1-2 did not influence autophagy or lysosomal activity. Instead, vtRNA1-2 is implicated in the regulation of angiogenesis, a key process in tumor progression. *VTRNA1-2*-promoter hypomethylation is correlated with chromatin accessibility in liver cancer samples and we uncovered an association between promoter methylation and key patient clinical conditions as registered in the TCGA metadata. These findings highlight a distinct oncogenic role for vtRNA1-2 in HCC and suggest that it may serve as a potential therapeutic target. Our study underscores the functional divergence among vtRNA paralogs, supporting the concept that each exerts unique biological effects rather than acting as redundant molecular entities.

## Graphical Abstract

**Graphical Abstract:**
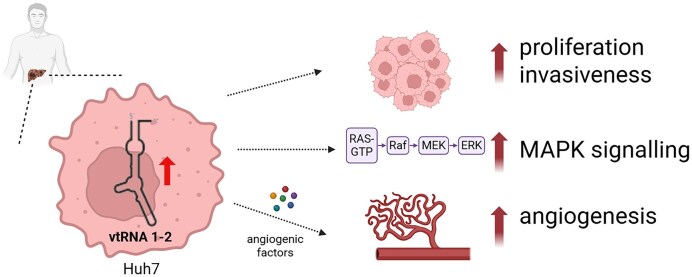

## Introduction

Vault RNAs (vtRNAs) are small noncoding RNAs (ncRNAs) and were initially identified as components of large ribonucleoprotein particles known as vaults [1]. Subsequent research revealed that a minor fraction of vtRNAs (<5%) is actually associated with the vault particle, hinting at broader, vault complex-independent functions [2, 3]. In the human genome, vtRNAs are located on chromosome 5q31 within two loci: VAULT-1, which includes three paralogs (*VTRNA1-1*,*VTRNA1-2*,*VTRNA1-3*), and *VAULT-2*, encoding vtRNA2-1 [4, 5]. In humans, these ncRNAs are transcribed by RNA polymerase III and range from 88 to 101 nucleotides in length [4, 6]. Recent research has revealed that vtRNAs play diverse roles in cellular processes such as immune response and drug resistance [7]. Additionally, vtRNAs have been implicated in regulating cell proliferation, apoptosis, lysosome biogenesis, and autophagy, and they may act as microRNA precursors [8–15]. These findings suggest that altered vtRNA expression is associated with tumorigenesis and chemotherapy resistance [11, 13, 16].

In addition to cell proliferation, ncRNAs can play crucial roles in cancer cell migration and invasion. By regulating the expression of genes involved in cell adhesion, cytoskeletal dynamics, and extracellular matrix (ECM) remodeling, ncRNAs facilitate the metastatic spread of cancer cells. For instance, miR-21 promotes tumor invasion and metastasis by targeting tumor suppressor genes, while long ncRNAs like H19 and MALAT1 modulate epithelial–mesenchymal transition and cell motility [17–19]. Among the various pro-proliferative functions attributed to ncRNAs in cancer cells, their role in promoting angiogenesis—the formation of new blood vessels from preexisting vasculature in surrounding healthy tissue—is particularly critical for supporting tumor growth and facilitating metastasis. Tumors require an adequate blood supply for oxygenation and nutrients, and ncRNAs have been found to be key regulators of this process [20]. Vascular endothelial growth factor (VEGF) is one of the primary mediators of angiogenesis, and ncRNAs can modulate its expression and activity. For example, microRNA miR-126 enhances angiogenic signaling and blood vessel formation, while miR-221 inhibits the angiogenic response by downregulating its target genes [21, 22]. Long ncRNAs also contribute to the angiogenic process; for instance, MALAT1 can promote endothelial cell proliferation and migration, enhancing blood vessel formation [23].

In this study, we aimed to elucidate the specific roles of vtRNAs encoded at the VAULT-1 locus in hepatocellular carcinoma (HCC), with a particular focus on their involvement in tumor cell proliferation, migration, and angiogenesis. By investigating the gene expression profiles and functional impacts of these vtRNAs, we sought to explore their potential as therapeutic targets, particularly in combination with established treatments. HCC is the primary malignancy of the liver, representing ∼75%–85% of all liver cancer cases globally [24, 25]. It originates from hepatocytes, the predominant cell type in the liver, and is characterized by aggressive growth and poor prognosis. HCC is the fourth leading cause of cancer-related deaths worldwide, with a particularly high incidence in regions with endemic hepatitis B and C virus (HBV and HCV, respectively) infections [26]. In addition, a quarter of the global population is estimated to have nonalcoholic fatty liver disease, which is the current fastest growing cause of HCC in the US and Europe [27]. HCC is often asymptomatic in its early stages, leading to late diagnosis and poor outcomes [28]. The management of HCC depends on the stage of the disease and includes surgical resection, liver transplantation, locoregional therapies (such as radiofrequency ablation and trans-arterial chemoembolization), and systemic therapies [29]. Among the systemic therapies, sorafenib (SF), a multi-kinase inhibitor, was the first approved treatment for advanced HCC and has demonstrated modest survival benefits [30]. Recent advancements in immunotherapy, particularly immune checkpoint inhibitors, have opened new avenues for treatment, offering hope for improved outcomes [31]. HCC remains a major global health challenge due to its aggressive nature and high mortality rate. Despite advancements in treatment, continued research is essential to develop novel medicaments and treatment approaches that can improve prognosis and survival rates of HCC patients. A deeper understanding of the molecular mechanisms underlying HCC will facilitate the development of targeted therapies, ultimately leading to improved clinical outcomes for those affected by this disease.

By combining whole transcriptome analyses with genetic, cell biology, and molecular biology approaches, we revealed a specific role of vtRNA1-2 for regulating cell migration, cell proliferation, and angiogenesis in HCC cells.

## Materials and methods

### Cell culture

The Huh-7 cells were cultured in Dulbecco’s modified Eagle’s medium (DMEM/F12; Thermo Fisher, 21331046) supplemented with 10% heat-inactivated fetal bovine serum (FBS; Thermo Fisher, 10082147) and 100 U/ml PenStrep glutamine (Thermo Fisher, 10378016). Huh-7 wild type (WT) and vtRNA1-1 knockout cells were kindly provided by M. Hentze (EMBL Heidelberg, Germany). On the WT cells, we generated a Huh-7 VTRNA1-2 knockout using CRISPR/Cas9 (see below). A single cell clone was obtained by serial dilution. We then derived a Huh-7 VTRNA1-2 complementation cell line using lentiviral transduction (see below) in the VTRNA1-2 knockout cells. After successful lentiviral transduction, positively infected cells were selected by treating them with 1 μg/ml puromycin for at least 2 weeks. A single cell clone was obtained by serial dilution. The EC-RF24 cells (kindly provided by P. Nowak-Sliwinska, University of Geneva, Switzerland) were cultured in 1:1 ratio DMEM/F12 and RPMI 1640 (Roswell Park Memorial Institute) medium (Thermo Fisher, 11875093) supplemented with 10% heat-inactivated FBS and 100 U/ml PenStrep glutamine. The HEK293T (kindly provided by O. Mühlemann, University of Bern, Switzerland) and HepG2 cells were cultured in DMEM/F12 supplemented with 10% heat-inactivated FBS and 100 U/ml PenStrep glutamine. All cells were incubated in a 5% CO_2_ environment at 37°C.

### Generation of vtRNA KO using CRISPR/Cas9

The *VTRNA1-2* gene was knocked out using the established and previously described protocol for generating vtRNA knockouts [9, 32]. To summarize, single guide RNAs (sgRNAs) targeting the up- and downstream regions of the VTRNA1-2 gene (Supplementary Table S1) were predicted using *crispr.mit.edu* online tool from the Zhang lab [33]. Matching oligonucleotides with 5′ BbsI overhangs were phosphorylated, annealed, and cloned into pCRISPR-EF1α-eSpCas9(1.1). These sgRNA constructs were tested for eSpCas9 cleavage efficiency by transfecting HeLa cells and analyzing genomic DNA with the GeneArt Genomic Cleavage Detection Kit (Thermo Fisher, A24372). Efficient sgRNAs were PCR (polymerase chain reaction) amplified with U6 promoter primers and cloned into pCRISPR-EF1α-eSpCas9(1.1) to create plasmids containing both upstream and downstream targeting sgRNAs and eSpCas9. The vtRNA1-2 locus, including the sequence between the targeted PAM sites, was PCR amplified and cloned into the genomic cleavage selection plasmid pMB1610-pRR-puro. Huh-7 cells were transiently co-transfected for 48 h with 0.5, 1.0, or 2.0 μg of pCRISPR-EF1α-eSpCas9(1.1) and 200 ng pMB1610-pRR-EF1α-puro, with the JetPEI DNA transfection reagent (Polyplus, 101000053). Subsequently, the cells were subjected to a 48 h selection period by replacing the medium with fresh medium containing 1.5 μg/ml puromycin. Single cell colonies were isolated, propagated, and total RNA was extracted for northern blot analysis.

### Lentiviral transduction

Lentiviral particles were generated in HEK293T cells, which were transiently transfected with lentiviral plasmids containing cDNAs coding for vtRNA1-2, together with the packaging plasmid pSPAX and the envelope plasmid pVSV-G (kindly provided by S. Geley, Medical University, Innsbruck). After 48 and 72 h, lentiviral supernatant was collected, sterile filtered (Sigma–Aldrich, Whatman Puradisc FP30, 0.2 mM), and supplemented with polybrene (Sigma, 107689) to a final concentration of 4 μg/ml and added to the Huh-7 VTRNA1-2 knockout cells overnight.

### Transfection and treatments

DNA plasmid transfections were done using Lipofectamine 3000 (Thermo Fisher, L3000008) according to manufacturer’s instructions. Locked nucleic acid (LNA) GapmeRs were transfected using Lipofectamine RNAiMAX (Thermo Fisher, 137780) according to manufacturer’s instructions. Antisense LNA GapmeRs (QIAGEN) were used to knock down vtRNAs. Two LNA targeting vtRNA1-1 as in [12] were used in equimolar combination, two LNA targeting vtRNA1-2 were used in equimolar combination, and the negative control (Supplementary Table S1) were used at 25 nM for 48 h. Sorafenib tosylate (MedChemExpress, HY-10201A) was used at 10 μM for the 30% inhibitory concentration (IC_30_). FITC-dextran (Sigma–Aldrich, FD40S) was directly diluted in the medium to 0.1 mg/ml.

### Cell proliferation and viability assay

Cell proliferation was quantified through automated cell counting. 0.1 × 10^6^ cells/well were seeded in six-well plates. After 24 , 48, and 72 h, cells were washed twice with phosphate buffered saline (PBS), detached using Accutase (Thermo Fisher, 00455556), and counted using a LUNA-II™ Automated Cell Counter (Logos Biosystems). Cell viability was measured with 3-(4,5-dimethylthazol-2-yl)-2,5-diphenyltetrazolium bromide (MTT; Thermo Fisher, M6494). 4.5 × 10^4^ cells/well were seeded into 24-well plates and allowed to attach overnight. After 24, 48, and 72 h, cell viability was assessed. Two hundred fifty microliters of the MTT solution (5 mg/ml of MTT in PBS) was added to each well that were incubated for 2 h at 37°C. The resulting formazan crystals were dissolved in dimethyl sulfoxide and the optical density was measured at 570 nm employing a Tecan infinite M1000Pro plate reader. Two independent experiments were conducted in duplicates.

### Colony formation assay

Approximately 5 × 10^2^ cells were seeded into each well of a six-well plate containing DMEM supplemented with 10% FBS and incubated for 10 days. Following incubation, colonies were fixed using pure methanol and stained with the crystal violet solution (0.1% crystal violet, 20% methanol) for 30 min. Three independent experiments were conducted in duplicates.

### RNA extraction and northern blot analysis

Cell total RNA was extracted with TRI Reagent (ZYMO Research, R2050-1-2000) with the conventional phenol–chloroform phase separation protocol as described [34]. Five micrograms of total RNA was separated on an 8% denaturing polyacrylamide gel (7 M Urea in 1× TBE buffer). The gel was subsequently electroblotted onto a nylon membrane (Amersham Hybond N+; GE Healthcare, RPN203B) as described [35]. The membranes were hybridized using ^32^P-labeled probes listed in Supplementary Table S1 and exposed to phosphorimaging screens for at least 24 h. An autoradiogram was developed using a Typhoon FLA1000 phosphorimager.

### Western blot analysis

The cells underwent lysis in RIPA buffer [50 mM Tris–HCl, pH 8.0, 150 mM NaCl, 1% Igepal CA-630, 0.5% sodium deoxycholate, and 0.1% sodium dodecyl sulfate (SDS)] supplemented with protease inhibitor cocktail (Sigma–Aldrich, 11697498001) and phosphatase inhibitors (Thermo Scientific, A32957). Protein extract concentration was determined with the Pierce BCA Protein Assay Kit (Thermo Fisher, 23227). Equal amounts of protein extracts (50 μg) were supplemented with 6× SDS loading dye (375 mM Tris–HCl, pH 6.8, 6% SDS, 30% glycerol, 30% β-mercaptoethanol, and 0.03% bromophenol blue) and separated on 10% denaturing SDS polyacrylamide gels and blotted onto pure unsupported nitrocellulose membranes 0.45 μm (PanReac AppliChem). Membranes were blocked for 1 h at room temperature in 5% skimmed milk powder in 1× TBST (200 μM Tris base, 1500 μM NaCl, 0.1% Tween 20) and then incubated in 3% bovine serum albumin fraction V in 1× TBST with the respective primary antibodies overnight at 4°C. The membranes were washed twice in 1× TBST and in 5% nonfat dry milk in 1× TBST were added horseradish peroxidase-conjugated secondary antibodies and incubated for 1 h at room temperature. The membranes were washed thrice in 1× TBST and the immuno-complexes were visualized using the SuperSignal West Femto Maximum Sensitivity Substrate (Thermo Fisher, 34096). The following primary antibodies and dilutions were used: rabbit anti-GAPDH 1:3000 (Cell Signaling, 14C10); mouse anti-p44/42 1:2000 (Cell Signaling, L34F12); Phospho-ERK1/2 Pathway Sampler Kit (Cell Signaling, 9911).

### Lysosomal pH measurements by flow cytometry

Lysosomal pH was measured using the protocol as described previously [36, 37]. Huh-7 cell lines were seeded and cultured for 72 h in cell culture medium containing 0.1 mg/ml FITC-dextran. After incubation, the medium containing FITC-dextran was removed and replaced with fresh medium, respectively, complete or starvation medium lacking FBS. The pH levels of lysosomes in both experimental-treated samples and those of the standard curve scale (with pH values ranging from 4 to 6) were examined using flow cytometry (FACS). Triplicate samples were analyzed for each condition.

### Vascular tube formation assay

The tube formation assay was performed as previously described [38]. In brief, the 96-well plates were first coated with 50 μl Matrigel matrix (Corning, 356234) per well. The plates were spun down at 50 × *g* for 5 min at 4°C and the Matrigel matrix was let to set on a leveled surface at 37°C for at least 1 h. In each coated well, 10 000 pretreated EC-RF24 cells were seeded and incubated for 4 h. The micrographs were captured on a microscope. The tube formation was analyzed using ImageJ. Triplicate samples were analyzed for each condition. The conditioned media from Huh-7 1-2 KO cells, Huh-7 WT cells, and HuH-7 1-2 complementation cells were collected for 24 h and applied to ECRF24 cells at a ratio of 60% conditioned media to 40% fresh media. The resulting vascular network formation was analyzed using the *Angiogenesis Analyzer* plugin in ImageJ.

### Cell migration and invasion assays

Cells were seeded in six-well plates for wound healing assays. Once the cells reached confluence, a straight scratch was made using 200-μl pipette tips. Images of the wound were captured at 0, 12, 24, and 36 h using a microscope. Four replicates were analyzed for each condition. Transwell invasion assays were conducted using a 24-well Transwell chamber with 0.8 μm pores (cellQART, 9328012). The chambers were precoated with Matrigel (Corning, 356234) according to the manufacturer’s instructions. Cells were suspended in 0.2 ml of serum-free medium and added to the upper chambers (1 × 10^5^ cells/well). The lower chambers were filled with medium supplemented with 10% FBS to serve as a chemoattractant. Cells were incubated for 24 h. After incubation, cells that had migrated to the lower membrane surface were fixed with methanol and stained with the crystal violet solution (0.1% crystal violet, 20% methanol). Invasive cells were counted in three randomly selected fields. The assay was performed two times independently, with experiments conducted in triplicate.

### VEGF ELISA on cell supernatant

Cells were incubated for 12 h at 37°C in hypoxic environment (1% O_2_). The supernatant medium was collected and filtered with 0.22-μm pore syringe filters (VWR, 514-1247). The hVEGF-A concentration in the media was measured using VEGF Human ELISA Kit (Thermo Fisher, KHG0111) according to manufacturer’s instructions. Absorbance was measured with Tecan infinite M1000Pro plate reader. The experiment was conducted in triplicate.

### Quantitative real-time RT-qPCR

To perform reverse transcription (RT) of total RNA, we utilized the SuperScript™ IV One-Step RT-PCR System (Invitrogen, 18090010) following the manufacturer’s instructions. This process involved the use of random primer hexamers for complementary DNA (cDNA) amplification. Subsequently, quantitative PCR (qPCR) was conducted on the synthesized cDNA using GoTaq® qPCR Master Mix (Promega, A6002) according to the manufacturer’s protocol. Details of the primers used are provided in Supplementary Table S1. qPCR amplification was performed using the Rotor Gene 6000 system (QIAGEN) as per the manufacturer’s instructions. Analysis of qPCR data was conducted using Roboticx software (QIAGEN), and differential mRNA transcript abundances were calculated using the 2^-ΔΔCT^ method, as previously described [39].

### Sequencing and data processing

The total RNA from WT, 1-1 KO, and 1-2 KO Huh-7 cells was extracted in triplicate. The libraries were prepared with the TruSeq Stranded mRNA kit (Illumina) and sequenced paired 2 × 50 bp on Illumina NovaSeq 6000.

The fastq files were aligned to a human reference genome hg38 version 104 with STAR (https://pubmed.ncbi.nlm.nih.gov/23104886/). The resulting sorted bam files were quantified with featureCounts (https://pubmed.ncbi.nlm.nih.gov/24227677/) to obtain the read count matrix. Differential expressed genes were identified using DESeq2 with an adjusted (false discovery rate, FDR) *P*-value threshold of .05. The list of differentially expressed genes (DEGs) is represented as volcano plots with the R package ggplot2. The list of DEGs was uploaded to Metascape for enrichment analysis with default parameters. The read count matrix was transformed to RPMs and log transformed *x*→log(1+*x*) and we performed a principal component analysis on the transformed data with the R function prcomp. Heatmaps were done with the heatmap.2 function of the R package gplots after obtaining the gene lists with the R function gconvert of the R package gprofiler2. Volcano plots were generated with a custom R script. Prior to plotting, 28 (KO 1-1) and 13 (KO 1-2) genes with adjusted *P*-value equal to 0 (below the computational precision limit) were excluded.

### Statistical analysis

Images were quantified using ImageJ. Data are expressed as mean ± standard deviation (SD). Statistical analysis was conducted with Student’s *t*-test, with *n* values specified in the figure legends. *P*-values <.05 are considered statistically significant. Graphs and plots were created using GraphPad Prism v10.

### ATAC-seq data analyses

The ATAC-seq data obtained for liver hepatocellular carcinoma (LIHC) samples from The Cancer Genome Atlas (TCGA) consortium were retrieved from the UCSC Xena Browser [40]. As is described in UCSC Xena Browser, to calculate the average ATAC-seq values, a prior count of 5 was added to the raw counts, then put into a “counts per million,” then log_2_ transformed, and quantile normalized; the result is the average value in the file (log_2_(count + 5) − qn values) across all technical replicates and all biospecimens belonging to the same TCGA sample group. The gene promoter for ATAC-seq is defined as a region within −1000 to +100 bp from the transcription start site (TSS). Peak location information for the ATAC-seq values of the vtRNA1-2 500 bp gene promoter regions was retrieved from the UCSC Xena Browser.

### DNA methylation data

The DNA methylation data and clinical information, including sample type, pathologic T, pathologic stage, residual tumor, overall survival (OS), and OS time (when available), were obtained from LIHC samples in TCGA consortium, retrieved from the UCSC Xena Browser [40]. It comprises the normalized beta-value of DNA methylation obtained using Illumina Infinium Human Methylation 450 BeadChip arrays. The normalized average promoter beta-values (500 bp bin) comprise the following CpG sites for vtRNA1-2: cg21161173, cg13303313, cg00500100, cg05174942, cg25984996, cg11807153, and cg15697852.

### Survival analysis based on DNA methylation

Samples were stratified into two groups (High and Low) based on the median methylation value of the vtRNA1-2 promoter in the LIHC cohort. Survival curves of the OS time and status values were generated using the Kaplan–Meier method (survival R package). Differences between groups were assessed using the log-rank test. The resulting survival curves were visualized using ggsurvplot from the survminer R package, which included 95% confidence intervals, risk tables, and *P*-values for statistical significance.

## Results

### The role of vtRNA1-2 in cell proliferation

**Figure 1.**
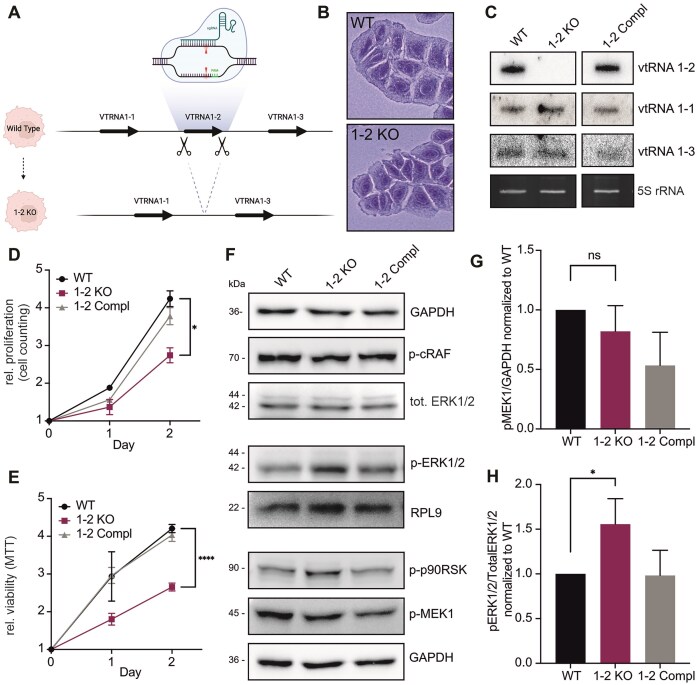
vtRNA1-2 knockout reduces viability and proliferation of HCC cells. (**A**) Illustration depicting the genomic editing approach for the elimination of the VTRNA 1-2 gene. (**B**) Microscopic images of cellular samples that were stained with Coomassie 24 h after seeding. (**C**) Northern blot analysis was employed to validate the efficacy of the knockout and complementation of the VTRNA1-2 gene in Huh-7 cells. 5S ribosomal RNA (rRNA) served as internal loading control. (**D**) The average proliferation rates, represented as mean ± SD, of Huh-7 WT, 1-2 KO, and complementation (Compl) cells were measured by automated cell counting. Values were normalized to day 0, *n* = 3. (**E**) The average viability rates, represented as mean ± SD, of WT, 1-2 KO, and 1-2 Compl cells were measured by the MTT assay. Values were normalized to day 0, *n* = 4. (**F**) Phosphorylation levels of proteins involved in the MAPK/ERK cascade, namely c-Raf, ERK1/2, p90RSK, and MEK, were evaluated after 20 min of starvation. GAPDH or RPL9 served as loading controls. (**G**) Quantification of p-MEK was conducted by normalizing the levels of p-MEK and total MEK with GAPDH, followed by calculating the ratio of p-MEK over total MEK. The values represent the mean ± SD of three independent experiments normalized to the WT level. (**H**) Quantification of p-ERK was conducted by normalizing the levels of p-ERK and total ERK with GAPDH (or RPL9), followed by calculating the ratio of p-ERK over total ERK. The values represent the mean ± SD of three independent experiments normalized to the WT level. Statistical significance was determined by *P-*values <.05, denoted in the results as follows: *P < .05; **P < .01; ***P < .001; ****P < .0001. *P-*values ≥.05 were considered not significant (ns).

In contrast to other vtRNA paralogs, vtRNA1-2 remains poorly characterized and its biological functions are largely unknown. To gain deeper insights into the molecular function of vtRNA1-2 within a controlled genetic context, we employed CRISPR/Cas9-mediated knockout of the vtRNA1-2 gene in the Huh-7 HCC cell line. Through precise targeting of the vtRNA1-2 gene using two sgRNAs, we achieved successful generation of vtRNA1-2 knockout (1-2 KO) cells (Fig. 1A). This approach led to the complete removal of the vtRNA1-2 transcriptional unit from the Huh-7 cell genome, providing a robust platform for investigating the specific roles and regulatory mechanisms associated with vtRNA1-2. Although the 1-2 KO cells did not exhibit pronounced morphological changes, subtle differences were observed. Particularly notable was the slightly jagged appearance of the cell edges compared to wild type (WT) cells (Fig. 1B). Using stable lentiviral transductions of the vtRNA1-2 gene, we successfully restored vtRNA1-2 expression in Huh-7 1-2 KO cells to levels comparable to those in WT cells, thereby generating a complementation (1-2 Compl) cell line (Fig. 1C). Removal of the vtRNA1-2 gene locus did not alter the expression of vtRNA1-1 or vtRNA1-3, the other vtRNA paralogs in the VTRNA1 locus (Fig. 1C). Cell proliferation was monitored over 48 h and 1-2 KO cells exhibited a significant growth reduction. However, this growth defect was effectively restored in the complemented cells, indicating the pivotal role of vtRNA1-2 in regulating cell proliferation (Fig. 1D). Similarly, the MTT metabolic activity supported these findings, revealing a significant impairment in the viability of 1-2 KO cells compared to the Huh-7 WT cells expressing vtRNA1-2 (Fig. 1E). The 1-2 KO cells exhibited a decrease in colony formation compared to the WT cells, indicating a compromised clonogenicity in the absence of vtRNA1-2 (Supplementary Fig. S1A and B). Together, these results highlight the pivotal role of vtRNA1-2 in regulating cellular proliferation and viability in HCC. In this respect, the 1-2 KO cells behaved similarly to the previously studied vtRNA1-1 knockout in Huh-7 cells [8].

### vtRNA1-2 regulates the MAPK/ERK cascade

In recent years, the intricate regulatory network involving vtRNA and the mitogen-activated protein kinase (MAPK/ERK) signaling pathway has garnered significant attention [8, 9]. Studies have highlighted the potential of the vtRNA1-1 paralog in modulating the MAPK/ERK pathway, thereby influencing various cellular processes such as proliferation, differentiation, and cell survival. Consequently, we examined whether the expression of vtRNA1-2 affects the MAPK/ERK cascade similarly as the vtRNA1-1 paralog. To address this, we monitored the phosphorylation status of the MAPK/ERK pathway in Huh-7 WT, 1-2 KO, and vtRNA1-2 complemented cells following a 20-min starvation. The loss of vtRNA1-2 did not result in an altered phosphorylation of cRAF nor the downstream MEK1 (Fig. 1F and G). However, the phosphorylation of ERK1/2 was significantly increased in the 1-2 KO cells compared to the WT cells. Notably, the ERK1/2 phosphorylation levels were restored in the complementation cells (Fig. 1F and H). A comparable phosphorylation pattern was previously observed in Huh-7 cells lacking vtRNA1-1, indicating that both vtRNAs likely modulate the MAPK/ERK cascade. Moreover, p90RSK, the effector downstream of ERK1/2 that regulates cell growth, proliferation, survival, and motility, was hyperphosphorylated in 1-2 KO cells compared to the controls (Fig. 1F). Together, these results highlight how vtRNAs collaboratively affect MAPK/ERK signaling, impacting cellular responses to environmental stimuli

### vtRNA1-1 and vtRNA1-2 KO transcriptome analysis

**Figure 2.**
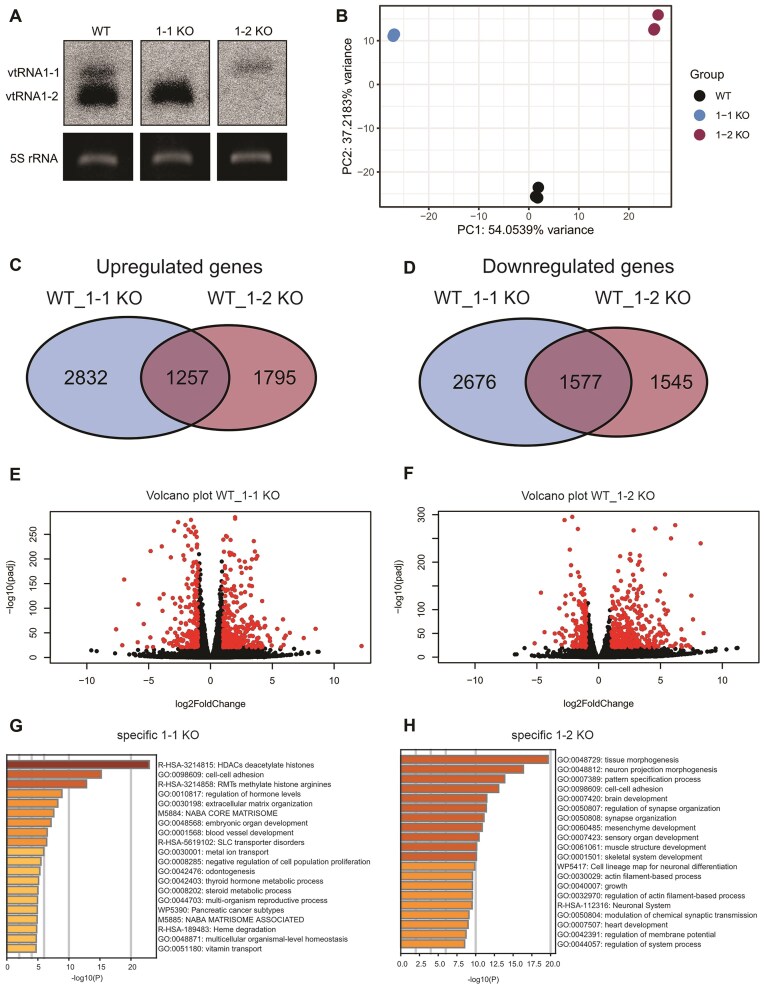
Sequencing of vtRNA KO cells reveals the affected cellular mechanisms. (**A**) Northern blot analysis of Huh-7 cells WT, 1-1 KO, and 1-2 KO used for high throughput sequencing. The levels of vtRNAs were assessed in cells cultured in complete media. 5S rRNA was utilized as an internal loading control. (**B**) Principal component analysis plot shows three biological replicates corresponding to the samples from Huh-7 cells in WT, 1-1 KO, and 1-2 KO. The RNA composition of cells differed significantly between the three conditions. (**C**) Venn diagram showing differentially expressed mRNAs between 1-1 KO and 1-2 KO, (**D**) differentially expressed downregulated genes between 1-1 KO and 1-2 KO. Significantly up- or downregulated mRNAs in the 1-1 KO (**E**) or in the 1-2 KO (**F**) cell lines relative to the WT control are depicted in red in the volcano pots. (**G**) Gene Ontology (GO) enrichment analysis of genes found with a log_2_ fold change >2 in WT compared to 1-1 KO sequenced samples. (**H**) GO enrichment analysis of genes found with a log_2_ fold change >2 in WT compared to 1-2 KO sequenced samples.

To decipher the function of vtRNAs in HCC cells, we performed mRNA sequencing (RNAseq) on vtRNA1-1 (1-1 KO), vtRNA1-2 (1-2 KO), and WT Huh-7 cells (Fig. 2A). The principal component analysis of the RNAseq data set showed tight clustering of the replicates and clear differences between all three cell lines (Fig. 2B). We next calculated the DEGs between 1-1 KO compared to WT control and 1-2 KO to WT control. There were 4089 DEGs upregulated in 1-1 KO compared to WT control and 3053 in 1-2 KO compared to WT control. Of those genes, 2832 DEGs were unique to 1-1 KO, 1795 unique to 1-2 KO, and 1257 DEGs were common for both (Fig. 2C). This apparent difference between the mRNA transcriptome of 1-2 KO and 1-2 KO cells indicates that the two vtRNA paralogs have discrete and unique functions. Likewise, there were both unique and overlapping downregulated DEGs in 1-1 KO and 1-2 KO cells compared to WT controls (Fig. 2D). The volcano plot revealed a pronounced bias toward upregulated genes particularly in 1-2 KO cells (Fig. 2E and F). RT-qPCR of the topmost up- and downregulated mRNAs in 1-2 KO cells validated these RNAseq-based analyses, with only a single exception (Supplementary Fig. S2). These observed differences in the of 1-1 KO and 1-2 KO cells indicated that the two vtRNA paralogs have direct influence on the transcriptome and most likely have distinct and unique functions.

As expected from previous studies [8, 9], the GO analysis confirmed that the regulation of cell growth is compromised in 1-1 KO cells. Furthermore, GO analysis revealed that the vtRNA KO cells might exhibit reduced competence in the cell motility compartment compared to the WT cells (Fig. 2G and H). Additionally, the GO analysis revealed that in vtRNA KO cell lines, tissue morphogenesis, cell–cell adhesion, and the *NABA matrisome-associated* genes are dysregulated compared to the paternal cell line. The latter GO term consists in a collection of genes encoding for ECM-associated proteins, including ECM regulators, and secreted factors. ECM components and matrisome-associated proteins play significant roles in the tumor microenvironment (TME) modeling and intercellular signaling pathways that regulate cancer cell behavior. They can bind to growth factors and cytokines, modulating their availability and activity, which in turn affects cellular processes such as angiogenesis, immune response, and cell differentiation [41–43].

In conclusion, our RNA sequencing analysis revealed that vtRNA1-1 and vtRNA1-2 paralogs have distinct and unique functions in HCC cells. GO term analyses suggest that both ncRNAs seem to be involved in regulating cell motility (among other cellular functions), which is crucial for the development and progression of HCC.

### vtRNA1-2 plays a pivotal role in regulating cell motility

**Figure 3.**
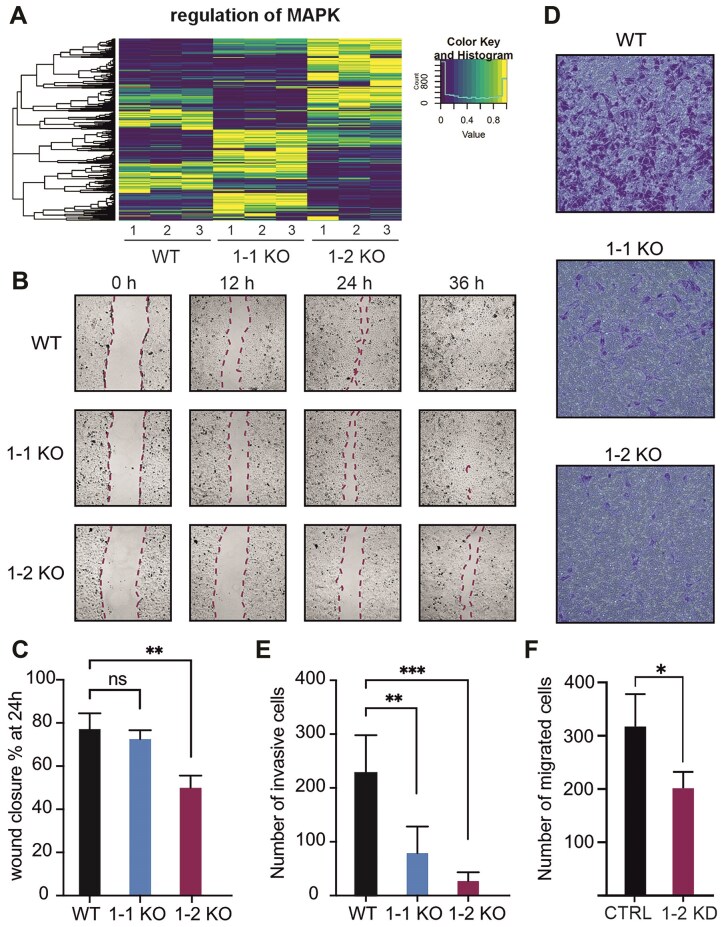
vtRNA enhances cell motility and invasiveness of HCC cells. (**A**) Heatmap showing the expression levels of a set of MAPK/ERK related genes. WT, 1-1 KO, and 1-2 KO have different MAPK/ERK related transcriptional landscapes. (**B**) Cells were seeded into 12-well plates and subsequently scraped using a pipette tip. Images were taken immediately after rinsing at 0, 12, 24, and 36 h. Data are representative of four experimental determinations. (**C**) The average percentage of wound closure was quantified after 24 h by measuring the area of the wounds ± SD (*n* = 4). (**D**) Cells were seeded onto Matrigel coated Transwell chambers. After 12 h of incubations, migratory cells were fixed and stained with crystal violet. Data shown are representative of six experimental determinations. (**E**) Migration of WT, 1-1 KO, and 1-2 KO cells through Transwell pores was quantified by counting the number of migratory cells under a light microscope. Data represent the means ± SD (*n* = 6). (**F**) Migration of Huh-7 cells upon knockdown (KD) of vtRNA1-2 through Transwell pores was quantified by counting migrated cells under a light microscope. A nontargeting LNA oligo served as negative control (CTRL). Data represent the means ± SD (*n* = 3). Statistical significance was determined by *P-*values <.05, denoted in the results as follows: *P < .05, **P < .01, ***P < .001, ****P < .0001. *P-*values ≥.05 were considered ns.

In accordance with Fig. 1F and our previous findings [8, 9], the RNAseq confirmed that the MAPK/ERK pathway was dysregulated in both 1-1 KO and 1-2 KO cells compared to the WT control (Fig. 3A). Intriguingly, the DEGs related to the MAPK/ERK pathway were distinct between the two vtRNA KO cell lines (Fig. 3A). This difference may explain why the two vtRNA KO cell lines exhibited a similar ERK1/2 phosphorylation pattern, yet displayed distinct cellular and molecular characteristics, as detailed below.

As indicated in Fig. 2G and H, the GO enrichment analysis suggested that pathways associated with cell migration and motility were negatively regulated in 1-1 KO and 1-2 KO cells. Therefore, we employed multiple strategies to investigate the cell motility of the HCC cells. Confluent monolayers of Huh-7 cells were subjected to scratch assays, and cells along the resulting wound were imaged by time lapse microscopy over a 36-h period (Fig. 3B). The WT cells exhibit a significantly higher migration speed compared to the vtRNA KO cells, resulting in a quicker healing of the wounded monolayers. The vtRNA1-1 knockout cells only showed a somewhat reduced migration speed, while the vtRNA1-2 knockout cells displayed a more pronounced and statistically significant impairment in migration (Fig. 3C). This difference was, however, not merely due to the known higher proliferation of WT cells compared to the 1-2 KO (Fig. 1D). In fact, to minimize the effect of cell proliferation, the experiment was conducted in reduced medium conditions. The effect of vtRNA on cell motility was confirmed by evaluating cell invasiveness through Matrigel-coated Transwell pores (Fig. 3D and E). WT cells demonstrated significantly higher motility compared to both vtRNA1-1 and vtRNA1-2 knockout cells. Particularly, Huh-7 cells lacking vtRNA1-2 expression showed a strong motility impairment. To validate these findings and to test whether these phenotypes were the result of clonal heterogeneities or peculiarities of the utilized Huh-7 WT and KO cell lines, the experiments were repeated under vtRNA1-2 knockdown conditions using LNA antisense oligonucleotides (ASOs). Following efficient and specific vtRNA1-2 knockdown (Supplementary Fig. S3A), these Huh-7 bulk cells behaved very similarly in both the scratch (Supplementary Fig. S3B) and the Transwell assays compared to the 1-2 KO cells (Fig. 3F). HepG2, another HCC cell line, showed comparable phenotypes in the MTT viability and the Transwell assays upon vRNA1-2 knockdown (Supplementary Fig. S3C and D), thus emphasizing that the observed vtRNA1-2-dependent phenotypes were not specific to one HCC cell line. Taken together, these experiments demonstrated that the expressions of both vtRNA1-1 and especially vtRNA1-2 promote migration and invasion of HCC cells.

### vtRNA1-2 does not regulate autophagy and lysosome biogenesis

Recent studies demonstrated vtRNA1-1 as a modulator of catabolic mechanisms in HCC [12, 44]. Recently, we revealed vtRNA1-1 as a positive regulator of autophagy, particularly by influencing lysosome biogenesis and activity [8]. Lack of vtRNA1-1 upregulated the phosphorylation levels of ERK, which consequently inhibited the nuclear translocation of the transcription factor EB (TFEB) [8]. TFEB is the master regulator of lysosome-related genes and therefore affects the maturation and function of lysosomes. Since the ERK phosphorylation pattern of 1-2 KO cells (Fig. 1F) resembled the one previously seen in 1-1 KO cells [8], we investigated whether vtRNA1-2 as well affects autophagy through the regulation of lysosomal activity. Looking into the sequencing data, we could confirm that autophagy and lysosome-related genes were differentially regulated in 1-1 KO cells compared to the WT cells (Supplementary Fig. S4A and B). However, the expression pattern of autophagy and lysosome-related genes in cells lacking vtRNA1-2 more closely resembled that of WT cells suggesting a different role for the two paralogs (Supplementary Fig. S4A and B). RT-qPCR analysis showed that the expression levels of TFEB and its target genes were not altered in the absence of the vtRNA 1-2 (Supplementary Fig. S4C). Taking advantage of the previously established *in cellulo* lysosomal pH measurement assay [8, 36], we demonstrated that the lysosomes of 1-2 KO cells, and in variance to the 1-1 KO cells [8], have a similar pH value as compared to the WT control (Supplementary Fig. S4D and E). These results clearly showed that, although both vtRNA paralogs have a similar impact on the MAPK/ERK cascade, they affect different downstream cellular processes. Unlike vtRNA1-1, vtRNA1-2 does not regulate autophagy and lysosome activity in Huh-7 cells.

### vtRNA1-2 is important for angiogenesis and tumor vascularization

**Figure 4.**
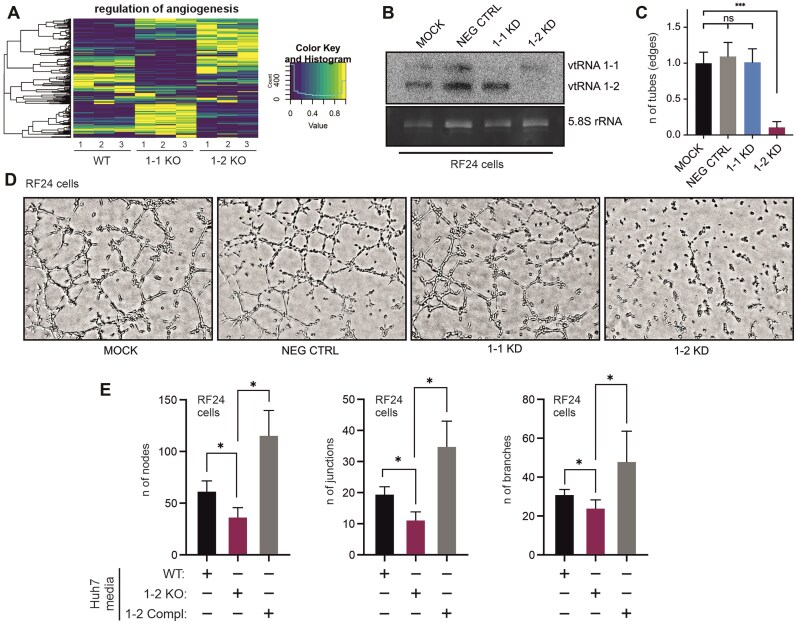
vtRNA1-2 regulates angiogenesis. (**A**) Heatmap showing the expression levels of a panel of genes associated with the regulation of angiogenesis. 1-2 KO cells display a highly different transcriptional landscape of this set of genes compared to WT and 1-1 KO samples. (**B**) Northern blot analysis was employed to validate the efficiency of ASO-mediated vtRNA knockdowns (KD) in EC-RF24 cells. 5.8S rRNA served as internal loading control. (**C**) The average number of edges formed during the tube formation assay ± SD normalized to the MOCK control (*n* = 3). (**D**) Endothelial cells NEG CTRL,1-1 KD, 1-2 KD, and the MOCK were seeded onto Matrigel coated wells. In negative control samples (NEG CTRL), a non-vtRNA-targeting ASO was used. After 4 h of incubations cells were imaged. Data shown are representatives of three experimental determinations. (**E**) Quantification of vascular tube formation network by RF24 cells incubated with conditioned media from Huh-7 WT, vtRNA1-2 KO, and vtRNA1-2 complementation cell lines. The average number of nodes, junctions, and branches formed during the tube formation assay was quantified. Data are presented as mean ± SD (*n* = 3). Statistical significance was determined by *P-*values <.05, denoted in the results as follows: *P < .05, **P < .01, ***P < .001, ****P < .0001. *P-*values ≥.05 were considered ns.

Tumor regulation of angiogenesis is a critical process in cancer progression, enabling tumors to secure an adequate supply of oxygen and nutrients for sustained growth and metastasis. This regulation involves various molecular signals and pathways, with VEGF-A playing a pivotal role in recruiting endothelial cells and promoting the formation of new blood vessels [45]. It has been extensively reported how ncRNA can regulate the TME and angiogenesis [45–48]. Therefore, we aimed to investigate whether this was the case for vtRNAs as well. According to our differential gene expression analysis, genes involved in the regulation of angiogenesis were dysregulated in Huh-7 cells lacking vtRNA compared to the WT cells. This disparity was particularly pronounced in the 1-2 KO cells (Fig. 4A and Supplementary Fig. S5). In patients, the vascularization of tumor masses is carried out by endothelial cells forming new blood vessels [49]. Since the mechanism of action of vtRNAs on angiogenesis is unknown, we decided to address this topic from both sides, from the HCC cell and the endothelial cell perspectives. Consequently, we first proceeded to examine the influence of vtRNA1-2 in EC-RF24 endothelial cells and their capacity for tube formation. Following the ASO-mediated knockdown of vtRNAs (Fig. 4B), their ability to form tubes were assessed. Indeed, vtRNA1-2 was found to play a pivotal role in tube formation, while an interconnected network of tubes was apparent in untreated endothelial cells and in the 1-1 KD cells. In clear contrast, essentially no tubes were formed as a consequence of the vtRNA1-2 knockdown (Fig. 4C and D). Second, to better understand the interconnection between vtRNA1-2 levels in Huh-7 cells on the one side, and the effect on tube formation in endothelial cells on the other side, this assay was repeated utilizing the secretome (conditioned media) of Huh-7 WT, 1-2 KO, and 1-2 Compl cell cultures on EC-RF24 cells (Supplementary Fig. S6A and B). Subsequently, the nodes, junctions, and branches in the tube formation assay were quantified. Significantly reduced numbers were observed in case conditioned media were used that originated from Huh-7 1-2 KO cells, whereas the media conditioned by 1-2 Compl cells fully rescued these defects in the tube formation assay (Fig. 4E). These data demonstrated that the secretome of HCC cells contains factors that trigger tube formation in epithelial cells, and this stimulatory effect on angiogenesis was dependent on vtRNA1-2 levels in both Huh-7 and RF24.

### Elevated vtRNA1-2 expression in liver tumor samples correlates with reduced overall patient survival

Our findings collectively implicated vtRNA1-2 as a crucial regulator of angiogenesis in liver cancer cell lines and possibly also in HCC. To investigate the relevance of vtRNA1-2 in LIHC, we analyzed its promoter methylation in the LIHC cohort from TCGA for its association with disease conditions and clinical staging. Since vtRNAs are not adequately sequenced by conventional RNAseq or small RNA-seq, but are known to be epigenetically regulated, we used the promoter methylation as a surrogate marker for their expression [16, 50]. Accordingly, *VTRNA1-2* and *VTRNA2-1* gene promoter accessibility measured by ATAC-seq is negatively correlated with their promoter DNA methylation across all TCGA cancer types (−0.74 *VTRNA1-2* and −0.78 *VTRNA2-1* Spearman correlation, *P*-value <.0001) [16]. Indeed, the latter report already showed that the *VTRNA1-2* promoter is significantly hypomethylated in tumor tissue compared to normal tissue of the LIHC-TCGA cohort.

**Figure 5.**
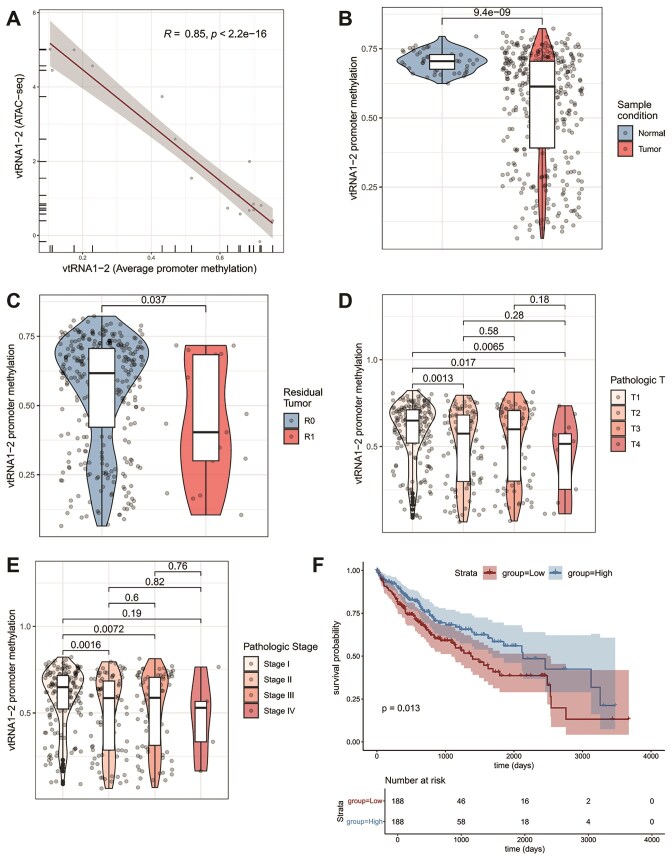
Association of *VTRNA1-2* promoter methylation with clinical parameters in the TCGA-LIHC cohort. (**A**) Scatter plot of the *VTRNA1-2* promoter DNA methylation and chromatin accessibility measure by ATAC-seq for samples (*n* = 17). The Pearson correlation was calculated for 17 tumors with available ATAC-seq and DNA methylation data (the Pearson *R* value and *P*-value are shown in the plot). (**B**) Box plot of the *VTRNA1-2* promoter DNA methylation of normal (*n* = 50) and tumor (*n* = 379) samples. (**C**) Box plot of the *VTRNA1-2* promoter DNA methylation of residual tumors R0 (*n* = 330) and R1 (*n* = 19) samples. (**D**) Box plot of the *VTRNA1-2* promoter DNA methylation of the different pathologic T classification (*n* = 376) samples. (**E**) Box plot of the *VTRNA1-2* promoter DNA methylation of the different pathologic stage classification (*n* = 355) samples. (**F**) OS analysis based on *VTRNA1-2* promoter methylation. Patients were stratified into two groups based on the median *VTRNA1-2* promoter methylation value (high versus low). OS was assessed using Kaplan–Meier analysis, and statistical significance was determined using the log-rank test. The association of *VTRNA1-2* promoter methylation with various available clinical parameters was evaluated using an unpaired two-tailed *t*-test.

To determine whether promoter methylation can serve as a proxy for vtRNA1-2 expression specifically in the liver, we first analyzed the correlation between ATAC-seq and DNA methylation values at the gene promoter. We found a significant inverse correlation between DNA methylation and chromatin accessibility at the *VTRNA1-2* promoter (*R* = 0.85, *P* < 2.2e−16), as measured by ATAC-seq in the 17 LIHC samples studied by ATAC-seq. This supports the use of promoter DNA methylation as a surrogate marker of vtRNA1-2 silencing in this tumor type (Fig. 5A). Given that ATAC-seq is available for only a subset of samples, DNA methylation arrays offer broader coverage across the TCGA cohort. We observed a significant reduction of *VTRNA1-2* DNA methylation in tumor samples (*n* = 379) compared to adjacent normal liver tissue (*n* = 50) (*P* < .0001) (Fig. 5B), suggesting that transcriptional activation of *VTRNA1-2* is associated with neoplastic transformation. We finally analyzed the association between *VTRNA1-2* promoter DNA methylation and key patient clinical conditions registered in TCGA metadata. A modest but statistically significant decrease in *VTRNA1-2* promoter methylation was apparent in patients with residual tumors (R1, *n* = 17) compared to those with complete resection (R0, *n* = 330) (*P* = .035) (Fig. 5C). This suggests a potential link between *VTRNA1-2* promoter methylation and surgical outcome. Additionally, significantly lower methylation was observed in more advanced tumors (T3–T4) compared to early stages (T1) (*P* < .01), as indicated by the pathological *T*-value, suggesting that the upregulation of *VTRNA1-2* expression via promoter demethylation correlates with tumor progression (Fig. 5D). Likewise, a significant reduction in promoter methylation was observed in higher pathological stages (Stage III–IV) compared to Stage I (*P* < .01) (Fig. 5E). Finally, a Kaplan–Meier survival analysis based on *VTRNA1-2* promoter methylation showed that patients with lower methylation levels (below the median), and thus elevated vtRNA1-2 expression, exhibit significantly worse OS compared to those with higher methylation (log-rank *P* = .03) (Fig. 5F). We noted that there was no association between the methylation of the paralogous *VTRNA2-1* promoter and the clinical characteristics of LIHC patients of the LIHC-TCGA cohort (Supplementary Fig. S7). This suggests a specific role of the vtRNA1-2 paralog in the disease. These observations highlight the potential prognostic relevance of *VTRNA1-2* epigenetic activation in HCC, thus suggesting a so far unknown link between elevated vtRNA1-2 levels and tumorigenesis in the liver.

Advanced HCC treatment typically involves the tyrosine kinase inhibitor SF, a drug known to impede tumor angiogenesis [51]. To explore the vtRNA-angiogenesis axis and to test the principal drugability of vtRNA, ASO-mediated vtRNA knockdown was combined with SF treatment in Huh-7. Indeed, lowering vtRNA1-2 levels in combination with SF significantly reduced cell viability in both Huh-7 and HepG2 (Supplementary Fig. S8A–D). These data underscore the potential of combining vtRNA-targeted ASO with established antiangiogenic drugs, such as SF, for modulating the TME to possibly potentiate therapeutic efficacy in HCC.

## Discussion

This study provides the first comprehensive analysis of the function of vault vtRNA1-2 in HCC cells. Our findings highlight the importance of vtRNA1-2 in cellular processes relevant for tumorigenesis and demonstrate its crucial role in cell proliferation, migration, and angiogenesis. Specifically, we observed a marked reduction in cell proliferation and viability (Fig. 1D and E), as well as impaired cell motility and invasiveness (Fig. 3 and Supplementary Fig. S1) in vtRNA1-2 knockout cells. These results are in agreement with a previous study in which the same cellular processes were affected by the absence of small RNAs derived from vtRNA1-2 in HEK cells [52]. These studies suggest that both the full-length vtRNA1-2 and its derived molecules can influence common pathways [52]. However, with the current state of research, it is challenging to determine whether these two RNA species have distinct or combined functions. Of note, though, Huh-7 cells expressed no detectable vtRNA1-2-derived small RNA species (Supplementary Fig. S9). Our results showed that vtRNA1-2, similar to its paralog vtRNA1-1, alter ERK1/2 phosphorylation levels thus regulating the MAPK/ERK signaling pathway (Fig. 1F and H). However, unlike vtRNA1-1, vtRNA1-2 does not affect autophagy or lysosome biogenesis (Supplementary Fig. S4), suggesting distinct regulatory functions between these two paralogs [8]. The difference between the vtRNA1-1 and vtRNA1-2 extend further, in Epstein–Barr virus-infected B cells. vtRNA1-1 was associated with the inhibition of intrinsic and extrinsic apoptosis, whereas vtRNA1-2 had no effect [10]. The expression of vtRNA1-1 modulates apoptosis via the amplification of the nuclear factor kappa-light-chain-enhancer of activated B cell (NF-κB) signaling cascade. In contrast, the NF-κB pathway is not affected by the vtRNA1-2 [10]. Along the same lines, we recently demonstrated a distinct dependency of vtRNA paralog stabilities on the newly identified vtRNA binding proteins TRIM21 and TRIM25 [53]. Some of the functional differences between the vtRNA paralogs can be attributed to structural variations between the transcripts. The three vtRNAs encoded at the VTRNA1 locus are similar in length and secondary structure. The double-stranded stem is ∼30 base pairs long and shares a similar sequence across the paralogs. However, the central loop domain differs in both sequence and length. The central loop is indeed the most divergent region among the vtRNA paralogs, and it is most certainly the primary reason these vtRNAs exhibit distinct cellular functions [9, 10]. This loop’s unique sequence and length are crucial for the specific interactions each vtRNA has with other cellular molecules, influencing pathways such as signal transduction, apoptosis, and gene expression regulation. Studies have shown that these differences in the central loop enable each vtRNA to bind different sets of proteins and RNA molecules, thereby modulating various cellular processes and contributing to their specialized roles within the cell. The functional diversity arising from these structural variations underscores the importance of vtRNA structure in determining cellular outcomes. [9, 44]. This divergence in function emphasizes the need for further investigation into the specific pathways governed by each vtRNA.

We showed here that, in contrast to vtRNA1-1, vtRNA1-2 plays a significant role in angiogenesis by its influence on tube formation in endothelial cells (Fig. 4A–D). Using conditioned media secreted by Huh-7 cell, either expressing high or low levels of vtRNA1-2, on seeded endothelial cells triggered or inhibited tube formation, respectively (Fig. 4E and Supplementary Fig. S6). These data suggest that Huh-7 cells secrete angiogenic factors, and this is in part dependent on vtRNA1-2 levels. One of the most obvious candidates for such a factor is VEGF-A. While we did not see changes in VEGF-A transcription in our RNA-seq dataset, reduced levels of secreted VEGF-A could be measured under hypoxic conditions in 1-2 KO cells compared to both WT and 1-1 KO cells (Supplementary Fig. S8E). More dedicated work is still required to comprehensively identify the angiogenic factors secreted by HCC cells.

Our study underscores the potential of vtRNA1-2 as a promising target for chemotherapy, particularly in HCC treatment. Targeting vtRNA1-2, either alone or in combination with existing drugs such as SF, could offer a novel therapeutic approach for HCC patients. The synergistic effect observed when combining vtRNA-targeting ASOs with SF (Supplementary Fig. S8A–D) indicated the potential for enhanced therapeutic efficacy in inhibiting tumor growth and angiogenesis. We like to point out that angiogenesis was impaired by reducing vtRNA1-2 levels either in HCC cells (Fig. 4E) or in endothelial cells (Fig. 4B–D), suggesting that the secretion of angiogenic factors by cancer cells and the responsiveness of ECs to those signals both depend on vtRNA1-2 concentration. Thus, our study provides valuable insights into the utility of vtRNA1-2 as a potential therapeutic target in HCC and endothelial cells, offering new avenues for the development of more effective treatment strategies. The distinct roles of vtRNA1-1 and vtRNA1-2 in HCC also suggest the possibility of differentially targeting these paralogs to achieve specific therapeutic outcomes. Investigating the expression patterns of these vtRNAs in various cancer types and stages could provide valuable insights into their role in cancer progression and resistance to therapy. Indeed, our unbiased association study of *VTRNA1-2* promoter methylation and liver cancer characteristics as well as clinical parameters utilizing the LIHC-TCGA cohort revealed significant correlations. *VTRNA1-2* promoter is significantly hypomethylated in tumor compared to normal tissue. Moreover, patients with elevated vtRNA1-2 expression, and thus reduced promoter methylation, exhibit a significantly reduced OS (Fig. 5). Overall, these findings position *VTRNA1-2* as a potentially valuable epigenetic biomarker of liver cancer with possible oncogenic roles as a cancer gene driver in the tissue.

In the mammalian kingdom, only the human and chimp genomes encode four vtRNAs. It is assumed that *VTRNA1-2* and *VTRNA1-3* genes originate from a recent gene duplication event [4]. For a long time, it remained unclear whether these paralogs serve a redundant physiological role or they have distinct functions. Our study points toward specific functions of vtRNA1-1 and vtRNA1-2 in human cancer cells. While both ncRNAs share pro-survival characteristics and both modulate the MAPK signaling cascade, they orchestrate distinct cellular mechanisms such as apoptosis and autophagy (vtRNA1-1 [8–10, 12]), or motility and angiogenesis (vtRNA1-2; this study). It remains to be determined whether different protein binding partners, distinct RNA folding architectures, or specific post-transcriptional modifications determine these functional characteristics of the vtRNA paralogs.

## Supplementary Material

zcaf028_Supplemental_File

## Data Availability

mRNA-seq data were deposited in the Gene Expression Omnibus database under accession number GSE276534.
