# Supplementary material for: Regulation of angiogenesis and cancer cell proliferation by human vault RNA1-2
Source: NAR Cancer. 2025 Aug 30;7(3):zcaf028. doi: 10.1093/narcan/zcaf028 (PMC12409401; doi:10.1093/narcan/zcaf028)
Supplement: zcaf028_Supplemental_File [file zcaf028_supplemental_file.pdf]

Supplementary Data

Regulation of angiogenesis and cancer cell proliferation by human vault RNA1-2

Stefano Gallo[1,2,†], Anastasiia Suspitsyna[1,2,†], Daniel Sanchez-Taltavull[3], Rafael S. Fort[4,5], Maria Ana Duhagon[3,6], Deborah Stroka[3], and Norbert Polacek[1,*]

[1] Department for Chemistry, Biochemistry and Pharmaceutical Sciences, University of Bern, Freiestrasse 3, 3012 Bern, Switzerland

[2] Graduate School for Cellular and Biomedical Sciences, University of Bern, Bern, Switzerland

[3] Department of Visceral Surgery and Medicine, Inselspital, Bern University Hospital and University of Bern, 3010, Bern, Switzerland.

[4] Sección Genómica Funcional, Facultad de Ciencias, Universidad de la República, Montevideo 11400, Uruguay

[5] Departamento de Genómica, Instituto de Investigaciones Biológicas Clemente Estable, Montevideo 11600, Uruguay

[6] Unidad Académica de Genética, Facultad de Medicina, Universidad de la República, Montevideo 11800, Uruguay

[†] Stefano Gallo and Anastasiia Suspitsyna contributed equally to this work

*Correspondence: Tel: +41 31 684 43 20; e-mail: norbert.polacek@unibe.ch

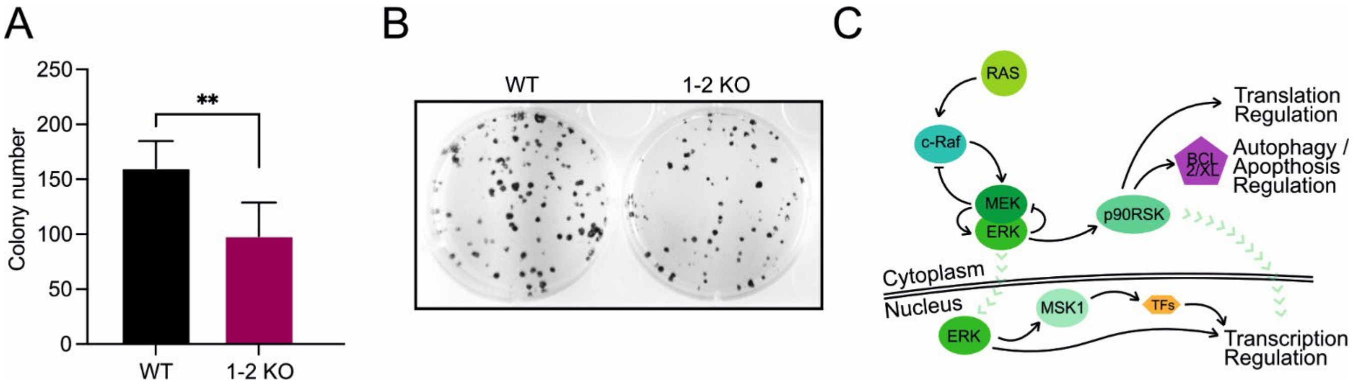

Supplementary Figure 1. (A) Clonogenic assay: data are expressed as the mean values of independent experiments ± SD, n=6. **(B)** Representative images of colony forming assay on WT, 1-2 KO and Compl, 2 weeks after seeding. **(C)** Schematic representation of the Mitogen-Activated Protein Kinase Pathway, the pivotal molecular effectors and cellular responses to the signals are included.

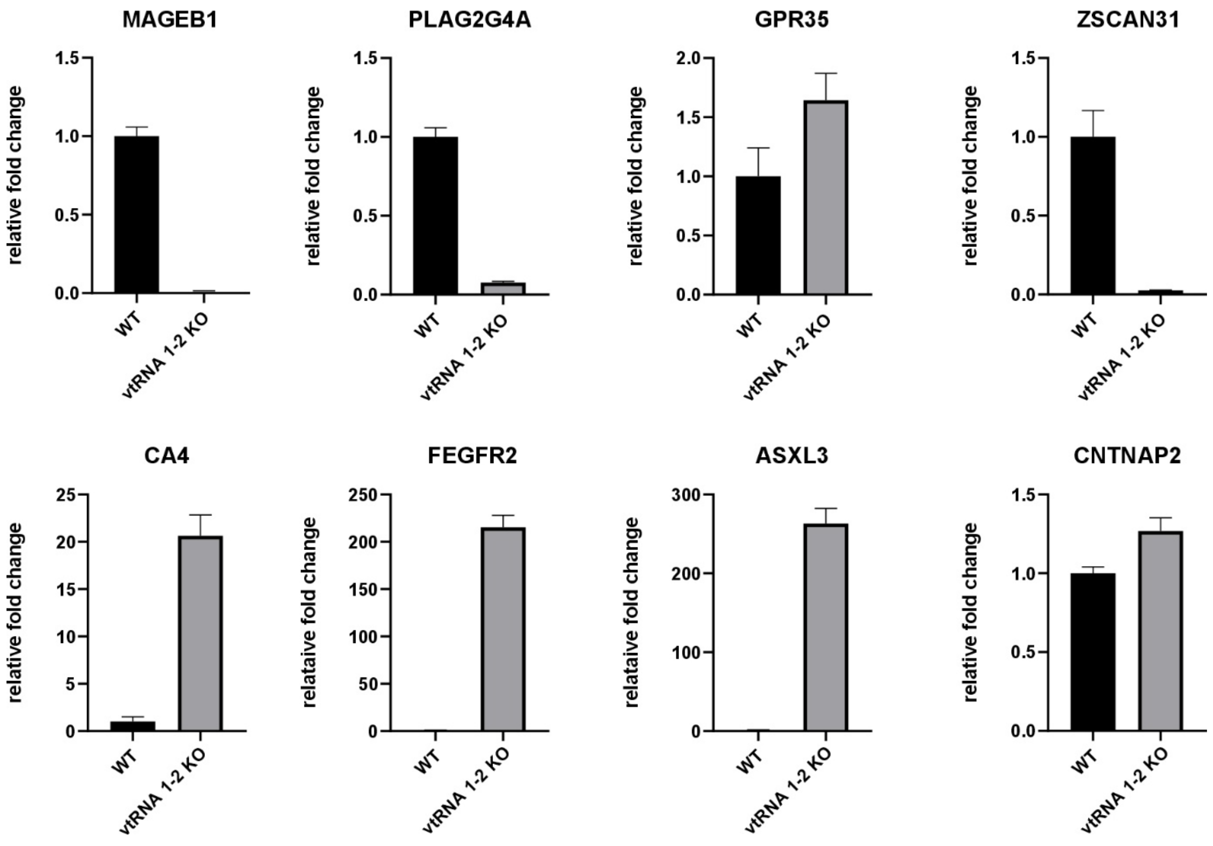

Supplementary Figure 2. Validation of the topmost up- and down-regulated mRNAs in Huh7 1-2 KO cells by RT-qPCR. In all cases gene expression levels were normalized to GAPDH mRNA levels.

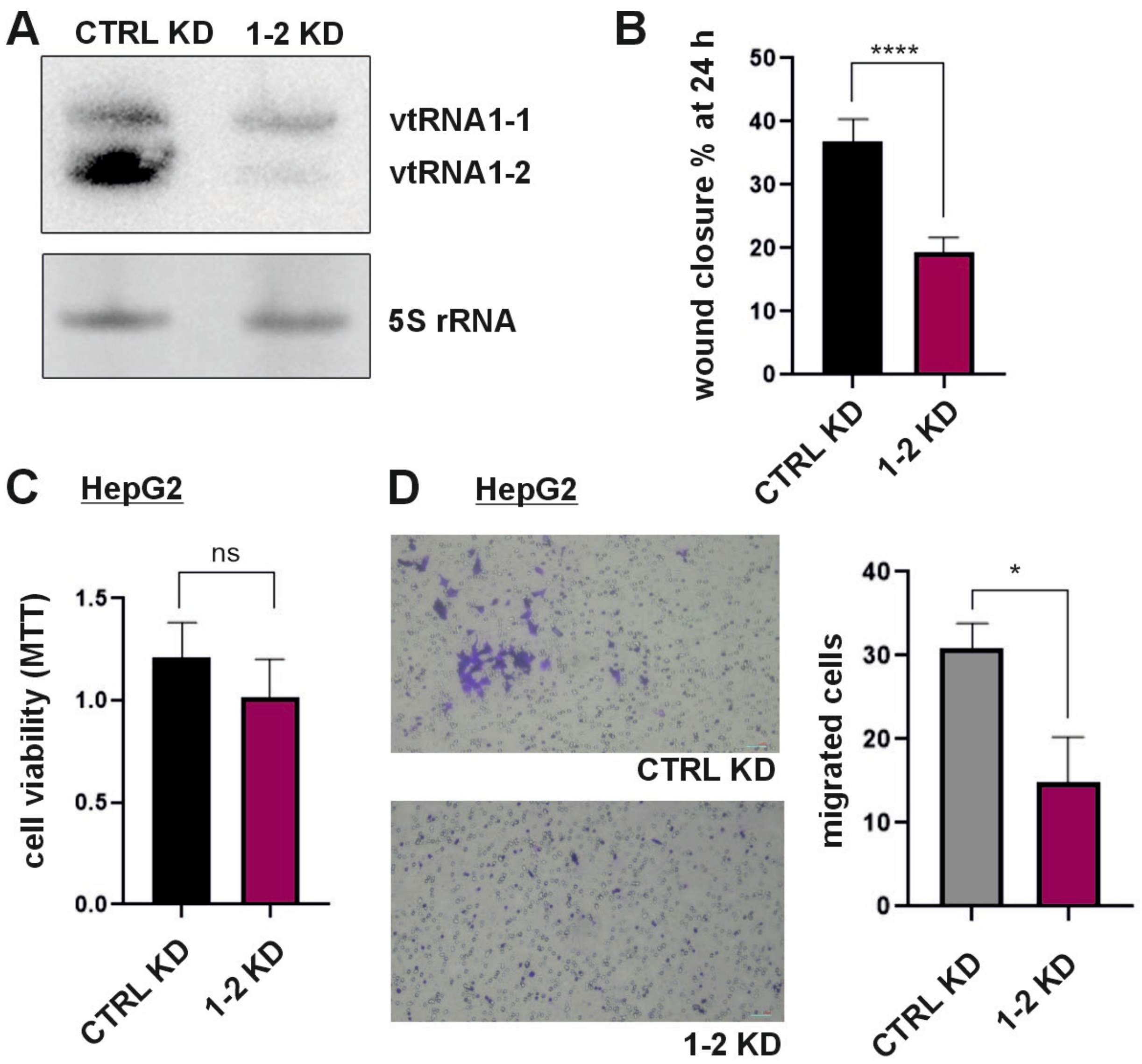

Supplementary Figure 3. (A) Confirmation of the Huh7 vtRNA1-2 knock-down (KD) by northern blot analysis. 5S rRNA (EtBr stained) served as loading control. **(B)** Huh7 1-2 KD cells in the scratch assay. The average percentage of wound closure was quantified after 24 hours by measuring the area of the wounds as mean and standard deviation (± SD; n=3). (**C)** The average cell viability of HepG2 cells in the absence (CTRL KD) or presence of reduced vtRNA1-2 levels (1-2 KD) was measured by the MTT assay on day 2 and is represented as mean ± SD. Values were normalized to day 1 (n=5). **(D)** HepG2 vtRNA1-2 KD cells in the MTT and Transwell assays. After 2 days of performing KD the cells were seeded on transwell plates. After 24 hours of incubations migratory cells were fixed and stained with crystal violet. Migrated cells were quantified by counting the number of cells under a light microscope. Data represent the mean ± SD (n=3). Statistical significance was determined by P values < 0.05, denoted in the results as follows: *P < 0.05; ****P < 0.0001. P values greater than or equal to 0.05 were considered statistically not significant (ns).

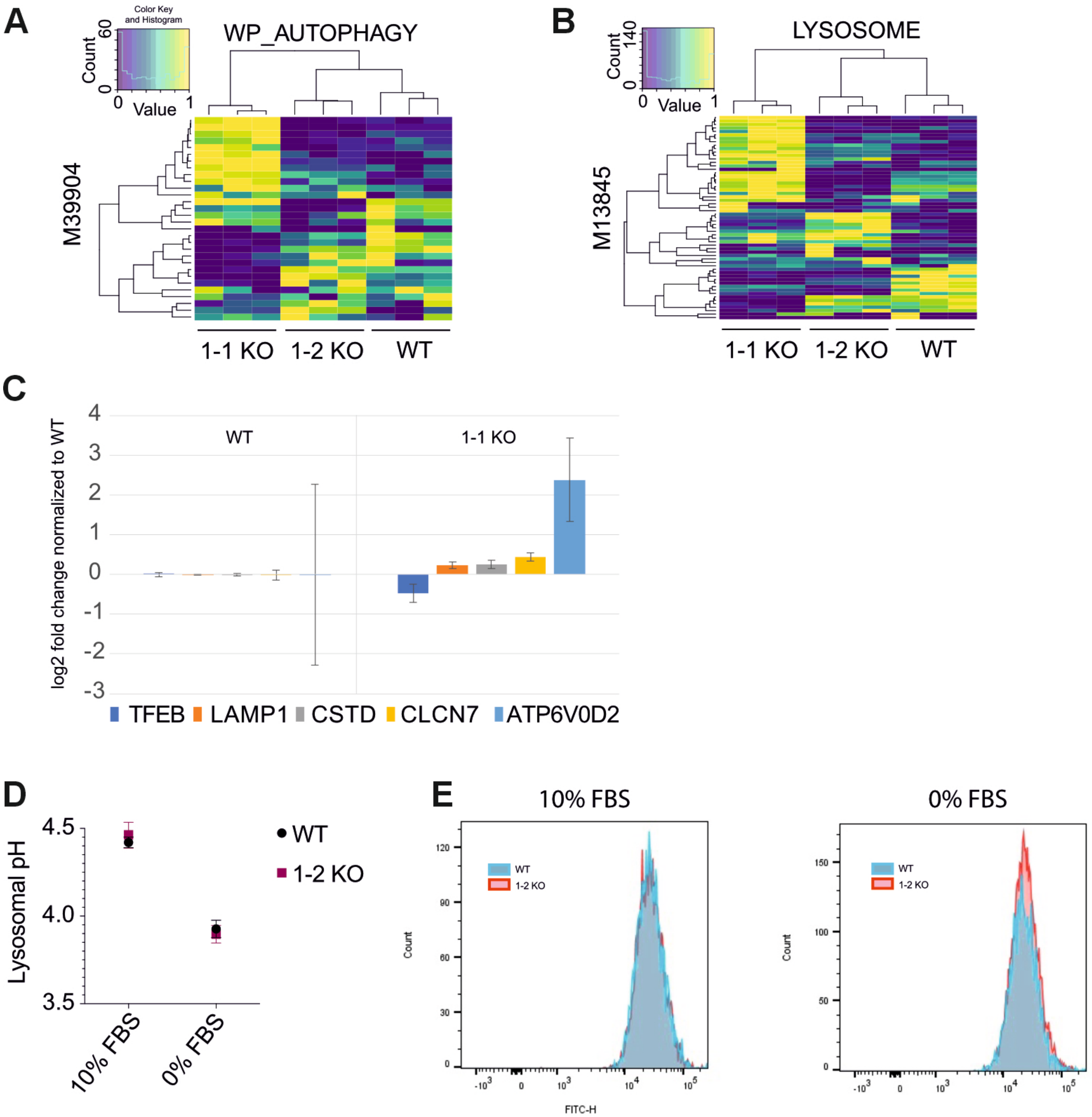

Supplementary Figure 4. Unlike vtRNA1-1 the vtRNA1-2 paralog does not regulate the cellular catabolism. **(A)** Heatmap showing the expression levels of a panel of genes associated to autophagy. As expected from previous study, 1-1 KO cells displays a highly different transcriptional landscape of this set of genes compared to WT samples. Notably 1-2 KO samples do closely resemble the transcriptional pattern of WT cells indicating that the lack of vtRNA1-2 does not affect the autophagy regulation in HCC cells unlike its paralogue. **(B)** Heatmap showing the expression levels of a panel of genes associated to lysosomes. As expected from our previous study, 1-1 KO cells display a highly different transcriptional landscape of this set of genes compared to WT samples. Notably 1-2 KO samples do closely resemble the

transcriptional pattern of WT cells indicating that the lack of vtRNA1-2 does not affect the autophagy regulation in HCC cells unlike its paralog. **(C)** Real-time qPCR data (mean ± SEM) of TFEB, LAMP1, CTSD, CLCN7 and ATP6V0D2 mRNA levels in Huh-7 WT and 1-2 KO cells grown in complete medium and starving culture conditions for 24 h, normalized to WT samples (n=3). **(D)** Mean ± SD of lysosomal pH values measured by flow cytometry in WT and 1-2 KO Huh-7 pre-incubated in culture medium supplemented with FITC-dextran (0.1 mg/mL, 72 h) left in complete medium (10% FBS) or followed by 6 h of starvation (0% FBS) in medium with low glucose, without amino acids and FBS (n=3). **(E)** Illustrative histograms from flow cytometry showing the overlap of FITC-dextran emission wavelength between WT and 1-2 KO samples.

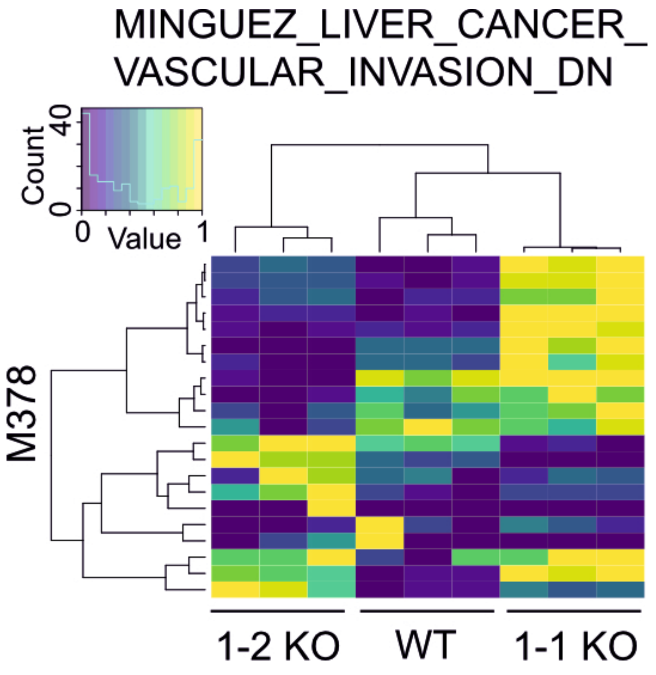

Supplementary Figure 5. Heatmap showing the expression levels of a panel of genes associated to liver cancer vascular invasion. 1-2 KO cells display a different transcriptional landscape of this set of genes compared to WT and 1-1 KO samples.

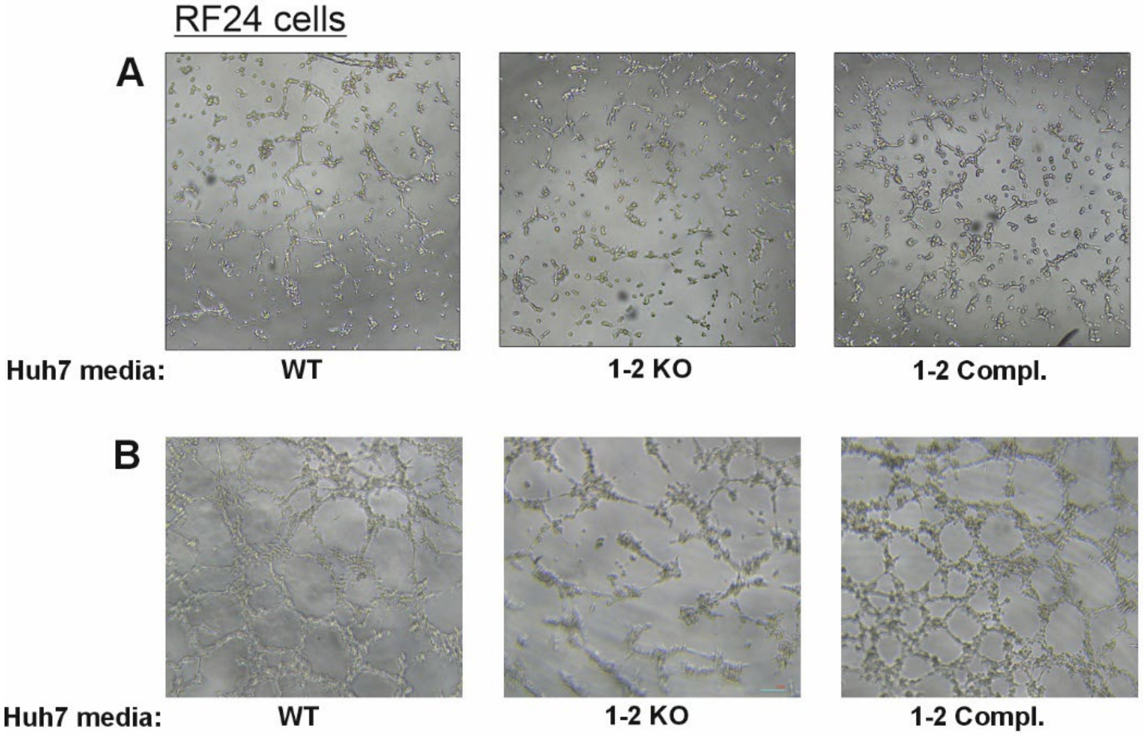

Supplementary Figure 6. Endothelial cells (EC-RF24) were seeded onto Matrigel coated wells and subsequently incubated with conditioned media obtained from Huh7 WT, 1-2 KO, and 1-2 Compl. cells. (**A)** 10,000 RF24 cells were seeded while in **(B)** 30,000 cells were seeded. The quantified data shown in the main Figure 4E originate from the seeding conditions as shown here in panel (A).

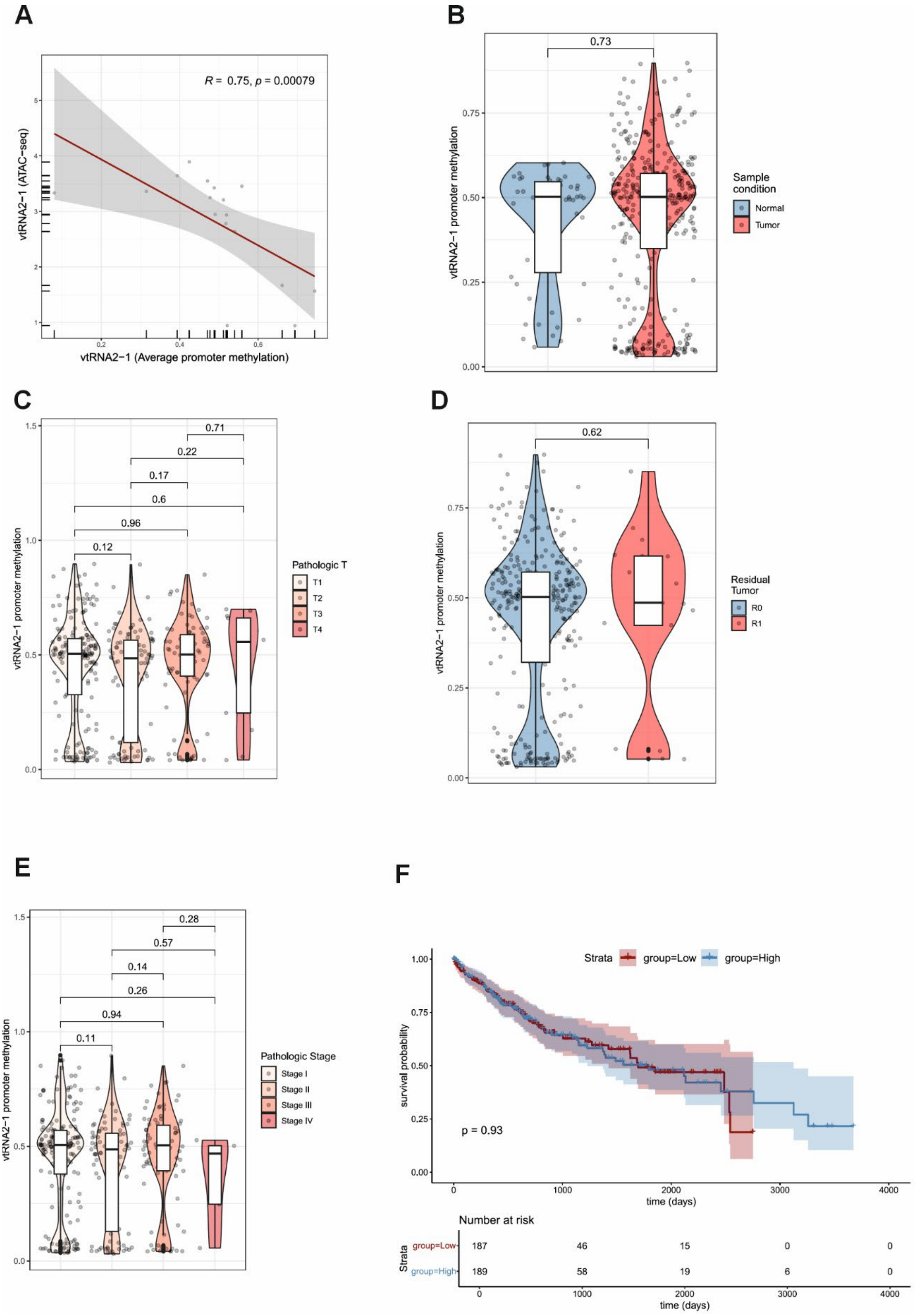

Supplementary Figure 7. No association of *VTRNA2-1* promoter methylation with clinical parameters in the TCGA-LIHC cohort. **(A)** Scatter plot of the *VTRNA2-1* promoter DNA methylation and *VTRNA2-1* promoter chromatin accessibility measure by ATAC-seq for samples (n=17). The Pearson correlation was calculated for 17 tumors with available ATAC-seq and DNA methylation data (the Pearson R value and

p-value are shown in the plot). **(B)** Box plot of the *VTRNA2-1* promoter DNA methylation of Normal (n=49) and Tumor (n=379) samples. **(C)** Box plot of the *VTRNA2-1* promoter DNA methylation of Residual Tumors R0 (n=330) and R1 (n=17) samples of TCGA-LIHC. **(D)** Box plot of the *VTRNA2-1* promoter DNA methylation of the different Pathologic T classification (n=375) samples. **(E)** Box plot of the *VTRNA2-1* promoter DNA methylation in samples with different Pathologic Stages (n=353). **(F)** Overall survival analysis based on *VTRNA2-1* promoter methylation. Patients were stratified into two groups based on the median *VTRNA2-1* promoter methylation value (high vs. low). Overall survival (OS) was assessed using Kaplan–Meier analysis, and statistical significance was determined using the log-rank test. The association of *VTRNA2*-1 promoter methylation with various available clinical parameters in the TCGA-PRAD cohort was evaluated: Normal vs. Tumor, Residual Tumor, Pathological T, and Pathological Stage, using an unpaired two-tailed t-test.

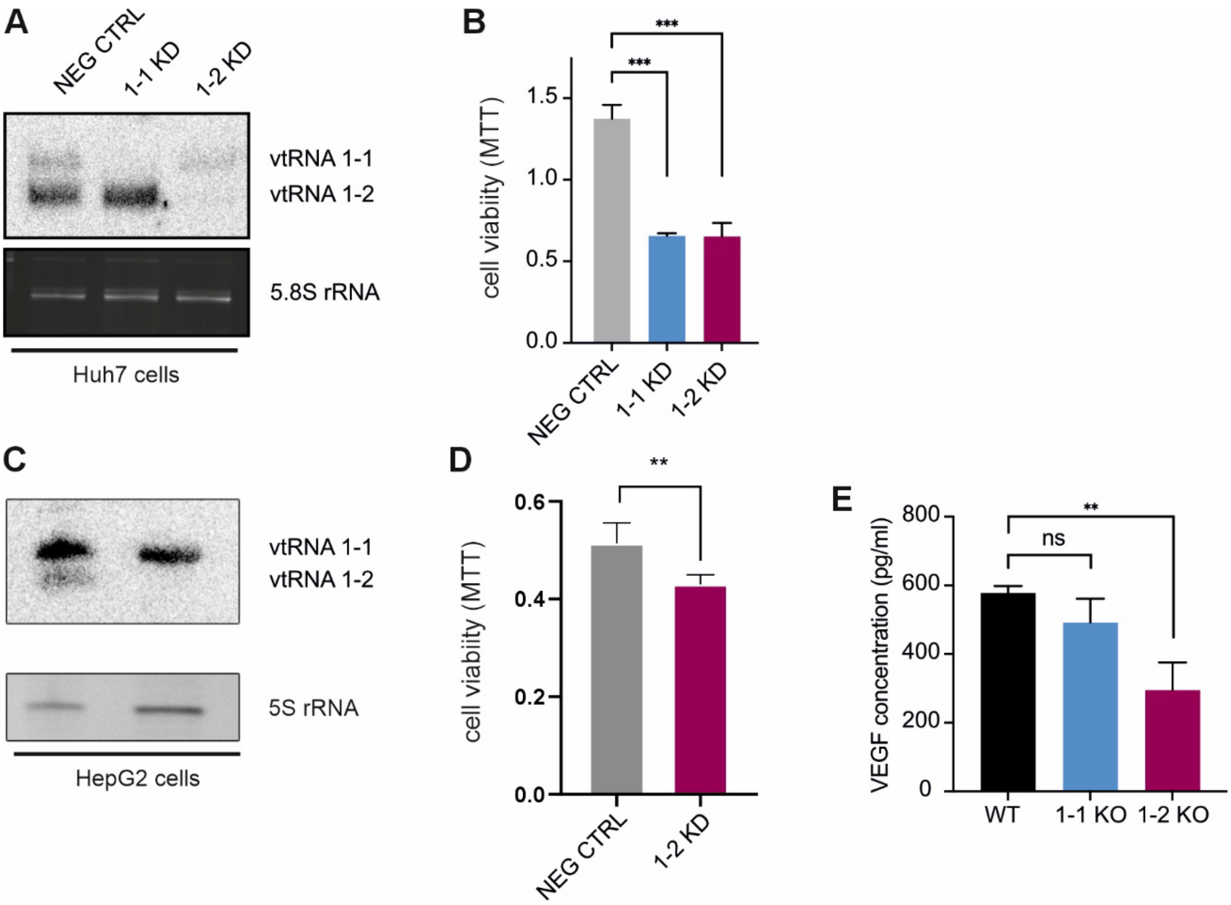

Supplementary Figure 8. Northern blot analysis was employed to validate the efficiency of vtRNA knock downs (KD) in Huh7 cells (**A**) or HepG2 cells (**C**). 5.8S or 5S rRNA was utilized as an internal loading control. **(B)** The average viability rates at day 3, represented as mean ± SD. In negative control samples (NEG CTRL) a non-vtRNA-targeting ASO was used. Huh7 NEG CTRL,1-1 KD, 1-2 KD cells were treated at day 0 with an IC30 dose of sorafenib and measured after 72 h by the MTT assay. Values were normalized to day 0 (n=3). **(D)** The average viability rates of HepG2 cells

at day 1, represented as mean ± SD. Cells were treated at day 0 with an IC30 dose of sorafenib and measured after 72 hours by the MTT assay. Values were normalized to the values of day 2 of untreated KD condition (n=5). **(E)** The average VEGFA concentration in the supernatant of WT, 1-1 KO, and 1-2 KO cells maintained in hypoxic environment for 12 h, represented as mean ± SD (n=3). Statistical significance was determined by P values < 0.05, denoted in the results as follows: *P < 0.05; **P < 0.01; ***P < 0.001; ****P < 0.0001. P values greater than or equal to 0.05 were considered not significant (ns).

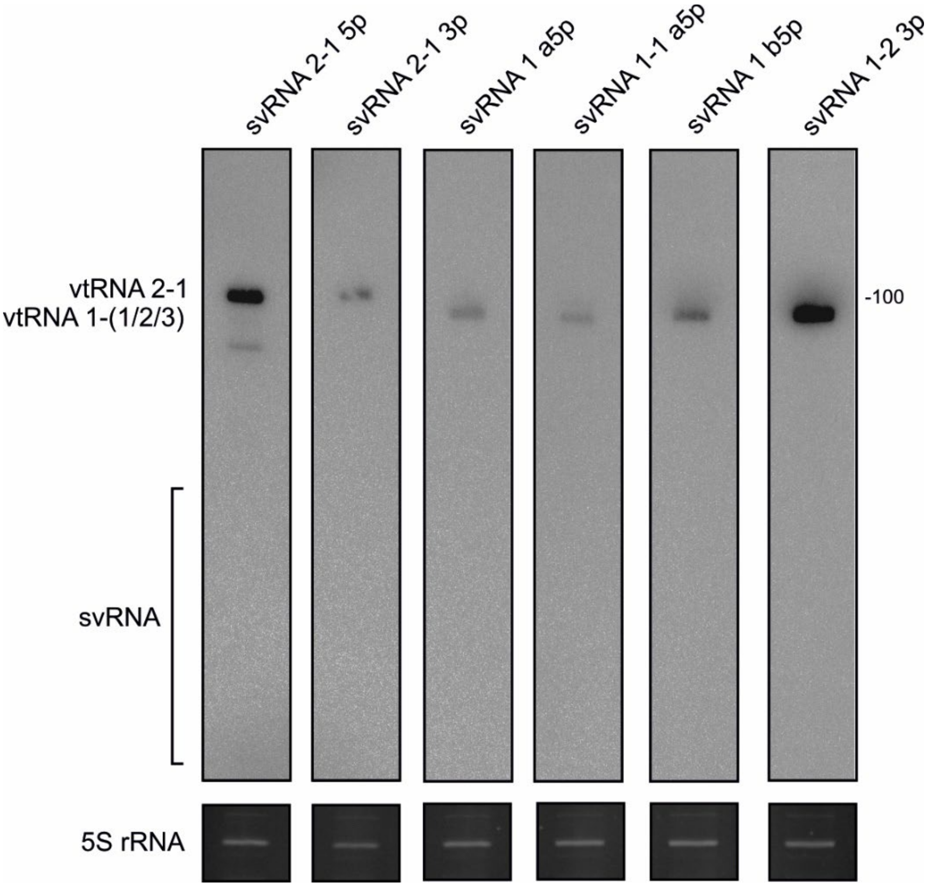

Supplementary 9. Northern blot analysis used to detect six previously described vtRNA-derived fragments. The probes can detect the full length vtRNA but failed to detect any smaller vtRNA-derived products (svRNA). The levels of RNA were assessed in cells cultured in complete media. 5S rRNA was utilized as an internal loading control.

Supplementary Table 1: DNA oligonucleotides used for constructing sgRNAs (i), performing northern blot analyses and PCR (ii), and for knocking down vtRNAs (iii)

(i) sgRNA ID	sequence 5' - 3'	PAM	
vtRNA1-2_Up 1	GGCTGTACACTCCAATTGTC	TGG	>
vtRNA1-2_Up 2	GACAATTGGAGTGTACACGCC	AGG	<
vtRNA1-2_Dw 1	GGGGTTTCACCGGGTTAGCG	AGG	<
vtRNA1-2_Dw 2	GGCGGGCGGATCACGAGGTC	AGG	>

(ii) Oligo ID	sequence 5' - 3'
vtRNA1-1_NB	GCTTGTTTCAATTAAAGAACTGTCG
vtRNA1-2_NB	AGGTGGTTACAATGTACTCGAAG
vtRNA1-3_NB	GAGGTGGTTTGATGACACGCGAA
svRNA2-1 5p_NB	CCGCTTGAGCTAACTCCGACCCG
svRNA2-1 3p_NB	AAGGGTCAGTAAGCACCCGCG
svRNA1 a5p_NB	ACCGCTGAGCTAAAGCCAGC
svRNA1-1 a5p_NB	CTGTCGAAGTAACCGCTGAGCTA
svRNA1 b5p_NB	TCGAAGTAACCGCTGAGCTA
svRNA1-2 3p_NB	AAAAGAGCTGGAAAGCACCCGCG
TFEB_Fwd	CCAGAAGCGAGAGCTCACAGAT
TFEB_Rev	TGTGATTGTCTTTCTTCTGCCG
CTSD_Fwd	AACTGCTGGACATCGCTTGCT
CTSD_Rev	CATTCTTCACGTAGGTGCTGGA
LAMP1_Fwd	ACGTTACAGCGTCCAGCTCAT
LAMP1_Rev	TCTTTGGAGCTCGCATTGG
ATP6VOD2_Fwd	TCTCACCTATATGACGTGCAGT
ATP6VOD2_Rev	GGTGGCACTTCCCCAGAATTT
CLCN7_Fwd	TGATCTCCACGTTCACCCTGA
CLCN7_Rev	TCTCCGAGTCAAACCTTCCGA
p62_Fwd	CATCGGAGGATCCGAGTGTG
P62_Rev	TTCTTTTCCCTCCGTGCTCC
Actin B_Fwd	CCAACCGCGAGAAGATGA
Actin B_Rev	CCAGAGGCGTACAGGGATAG
MAGEB1_fw	TCGTCCCAGGCTGCTAGATA
MAGEB1_rev	AGCTTGTGGGAGTATCCCCT
ZSCAN31_fw	GGTTG ATCGGTCATTGGGGT
ZSCAN31_rev	AGTTGTCGGGAGGCTTCTTG
PLAG2G4A_fw	GCTAGAGGCATTGAGGAGCC
PLAG2G4A_rev	CCTGCTGTCAGGGGTTGTAG
GPR35_fw	CCAGCCCTTCTCAGACAGC
GPR35_ rev	CATGTAGATGCGGGTCTCCG
CNTNAP2_fw	AAGCCCTTACACTTGGTGGG
CNTNAP2_rev	ATTCCTGTCTCCTTGGCAGC
FGFR2_fw	CGCTGGGGAATATACGTGCT

FGFR2_rev	CTGTTACCTGTCTCCGCAGG
ASXL3_fw	AAGAAGAAGGACCGCACCTG
ASXL3_rev	GGGCATGACGACTCCTCTTT
CA4 fw	CCTTGGTGGTGACGATGTTGAT
CA4_rev	CACTGGTGCTACGAGGTTCA

**(iii) LNA GapmeR ID**	sequence 5' - 3'
vtRNA1-1_LNA_1	TTAAAGAACTGTCGAA
vtRNA1-1_LNA_3	TTAAAGAACTGTCGA
vtRNA1-2_LNA_1	TACTCGAAGTAACCGC
vtRNA1-2_LNA_3	TGGAAAGCACCCGCGG
Neg_CtrlA	AACACGTCTATACGC
